# Advances in Mechanical Circulatory Support (MCS): Literature Review

**DOI:** 10.3390/biomedicines13071580

**Published:** 2025-06-27

**Authors:** Jasmine K. Dugal, Arpinder S. Malhi, Yuvraj Singh, Rooz Razmi, Joshua Vance, Divyansh Sharma

**Affiliations:** 1 Department of Internal Medicine, Kirk Kerkorian School of Medicine, University of Nevada Las Vegas, Las Vegas, NV 89102, USA; arpinder.malhi@unlv.edu; 2 Lake Erie College of Osteopathic Medicine (LECOM), Erie, PA 16509, USA; ysingh46067@med.lecom.edu; 3 Michigan State University College of Osteopathic Medicine (MSUCOM), East Lansing, MI 48824, USA; razmiroo@msu.edu; 4 A.T. Still University Kirksville College of Osteopathic Medicine, Kirksville, MO 63501, USA; joshua.vance@atsu.edu; 5 Department of Cardiology, Kirk Kerkorian School of Medicine, University of Nevada Las Vegas, Las Vegas, NV 89102, USA; divyansh.sharma@unlv.edu

**Keywords:** heart failure, cardiogenic shock, left-ventricular assist device, Bi-ventricular assist device, impella, total artificial heart, intra-aortic balloon pump, extra-corporeal membrane oxygenation, survival rates, clinical outcomes, MCS complications, bridge to transplant, destination therapy

## Abstract

Heart failure is a heterogeneous disorder that can lead to cardiogenic shock. Mechanical circulatory support (MCS) devices can replace the cardiac function in an attempt to bridge patients to transplant or they can serve as destination therapy to improve overall patient functionality and in turn quality of life. Currently utilized MCS devices include devices such as Left Ventricular Assist Devices (LVADs), Biventricular Assist Devices (BiVADs), Impella, Total Artificial Hearts (TAHs), and extracorporeal membrane oxygenation (ECMO). This literature review examines a range of studies, case reports, and meta-analyses to present current approaches to mechanical circulatory support (MCS), along with the challenges and limitations of existing devices, common complications, and overall survival and long-term outcomes following MCS therapy.

## 1. Introduction

### 1.1. Overview of Heart Failure

Heart failure (HF) is characterized by inefficient myocardial function, impacting ventricular filling and/or ejection of systemic blood flow. As a result of the lack of blood distribution, the body’s metabolic demands are not met, leading to several associated symptoms and potentially cardiogenic shock (CS). These symptoms can include, but are not limited to, chest pain, shortness of breath, swelling of the lower extremities, and in later stages, death [1]. HF can be caused by several conditions and factors, all leading to a pathologic circulatory system. HF is a condition that is ubiquitously seen throughout the world, affecting millions of individuals. Globally, HF is a leading cause of hospitalization among individuals aged 65 and older, contributing to increased morbidity and mortality rates. In 2012, HF-related healthcare expenditures in the United States were estimated at $31 billion, accounting for over 10% of total cardiovascular disease costs. Projections indicate that these costs could increase by 127% by 2030 [2]. Experts describe HF as a global pandemic, affecting 26 million people throughout the world. As the population ages, healthcare costs and morbidity have increased, presenting an unfortunate fate for those affected [3]. Due to the implications it has on healthcare costs, life quality, and patient life spans, HF is truly a pressing issue in regards to the general health of the entirety of humanity.

### 1.2. The Role of Mechanical Circulatory Support (MCS)

In modern medicine, methods of HF treatment have developed, which involve technology to aid in the patient’s circulatory system. Mechanical Circulatory Support (MCS) involves the use of various devices to monitor and manage cardiac function when in need of HF treatment. These devices replace the ineffective heart’s pumping mechanism to support cardiac recovery, stabilize patients awaiting definitive heart transplantation, and maintain proper circulation and symptom relief in patients who cannot maintain functional capacity. MCS technology has evolved from being a cumbersome and short-term circulatory aid to an efficient and permanent HF treatment. As a result of these developments, certain MCS devices now have the potential to perform as long-term alternatives for patients unsuitable for transplantation, having significantly increased clinical indications from what was previously only a temporary form of bridging treatment. MCS technology is now a crucial component of all-encompassing HF care, greatly enhancing clinical results, patient survival, and quality of life.

### 1.3. Objectives of the Paper

Advancements to MCS in the present have had a net positive effect on the outcomes of patients who suffer from HF. Not only are HF patients who do not qualify for transplantation living longer, but they are also living with a higher quality of life. These positive effects can be attributed to the miniaturization of devices such as Left Ventricular Assist Devices (LVADs), Biventricular Assist Devices (BiVADs), and Total Artificial Hearts (TAHs). Technological innovation results in smaller, more portable MCS systems that allow greater patient mobility and compatibility [4]. Furthermore, through the use of modern materials, the biocompatibility of patients to MCS devices has increased, reducing the risk of complications such as thrombosis and device failures [5]. The future of MCS is set to improve the technology that has been developed via further miniaturization, changes in device design and uses, and the integration of remote monitoring of vitals and conditions. MCS technology is continuing to evolve in the present.

## 2. Current Approaches in Mechanical Circulatory Support

### 2.1. Types of MCS Devices

#### 2.1.1. LVADs

LVADs are implanted mechanical pumps that support left ventricular (LV) function in cases of purely or predominantly left-sided HF. In these devices, the primary driver of blood flow is the ventricle, with some assistance from the LVAD. During implantation, an inflow cannula is inserted through the apex of the heart. This directs blood from the left ventricle (LV) to an electric motor, then on through a tube to the aorta. The motor is powered by a drive line connected to a small, external controller, which is, in turn, connected to an external battery. This allowed patients to receive continued hemodynamic support outside of the hospital. Previous iterations of LVADs used pulsatile motors in an attempt to mimic normal physiology; more modern devices, however, use continuous flow (CF-LVADs) with artificial pulsatility, as they tend to be more durable [6].

In patients with irreversible dysfunction with refractory symptoms of HF despite optimized medical therapy, LVADs are the best treatment option besides heart transplantation (HTx) [7]. A recent meta-analysis of patients who received LVADs shows a significant increase in survival rates at 6, 12, and 18 months compared to other treatment options (OR 1.96) [8]. LVADs are, therefore, often used as a bridge to transplantation (BTT) for HTx. Because of their efficacy, they are sometimes also used as destination therapy (DT) [9].

Despite their benefits, LVADs have several drawbacks. While patients with this device have better survival, they are significantly more likely to be rehospitalized for complications (OR 2.98), including adverse events such as bleeding, infection, stroke, and arrhythmia [8]. Notably, LVADs often provide inadequate support for LV dysfunction due to arrhythmias, and surgical implantation can, indeed, cause arrhythmias. Likewise, while they can reduce symptoms of RH failure secondary to LH failure, LVADs are inadequate in patients with primary RH dysfunction [7].

Notable devices in use include HeartMate 3 and HeartMate II by Abbott and HeartWare HVAD by Medtronic (though HeartWare was removed from the market in 2021) [10].

#### 2.1.2. BiVADs

BiVADs are the configuration of two ventricular assist devices (VADs) for the purpose of supporting both left and right ventricles in the setting of severe dysfunction. The BiVAD configuration is achieved by using both a temporary RVAD and LVAD or by using two LVADs [11]. BiVAD can serve as an important bridge to transplantation (BTT), but it is associated with significant mortality. Where LVADs have a 1-year survival rate of 82%, BiVADs are only 56% at 6 months [12,13]. Part of this is related to the greater iatrogenic damage associated with installing two devices rather than one.

In recent years, there has been a significant shift away from pulsatile VADs to continuous-flow VADs. However, there is currently no on-label continuous-flow RVAD available to treat chronic right heart failure (RHF) aside from Total Artificial Hearts (TAH), discussed below. All other on-label RVADs are temporary devices such as the RP Impella used in isolated RHF. This is a problem for several reasons. Firstly, up to 40% of patients who receive an LVAD subsequently develop RHF [14]. Many of these then require RVAD, which shows significantly greater mortality than when BiVAD is implanted concomitantly [15]. Secondly, the lack of therapy options has led to a variety of methods to modify LVADs for right-sided use [16]. Recent small studies of BiVAD using dual HeartMate3 implants have shown large differences in survival between centers—for instance, one study showed a survival rate of 91% at 18 months, and another showed a rate of roughly 54% [17,18]. The discrepancy may, of course, be ascribed to several confounding variables (e.g., support methods, sample size), but as there is a paucity of randomized controlled trials for BiVADs, the variety of LVAD modification techniques might very well play a part in the poor outcomes associated with BiVAD use.

#### 2.1.3. Impella

Percutaneous MCS, such as the Impella (Abiomed, Danvers, MA, USA) microaxial pump, has been demonstrated to enhance peripheral organ perfusion, lower myocardial oxygen demand, and raise coronary perfusion. Thus, preemptive implantation during high-risk percutaneous coronary intervention (HR-PCI) and emergency use for CS (CS) are among the indications for the Impella device [19]. The Impella is a continuous-flow device that actively translocates oxygenated blood from the LV to the ascending aorta regardless of the cardiac phase and function. Current Impella devices on the market include the Impella 2.5, Impella CP, Impella 5.0 and 5.5, and the Impella RP model for right ventricle (RV) support. Of these, the Impella 2.5 and CP can be inserted percutaneously, while the 5.0 and 5.5 require surgical placement. Similar to how an intra-aortic balloon pump (IABP) is placed, the Impella 2.5 and Impella CP variants are often placed using a retrograde femoral arterial route. An arteriotomy is necessary for the implantation of the larger Impella 5.0 device. By using an end-to-end anastomotic conduit, the Impella 5.0/LD can be inserted straight into the proximal aorta. With the aid of fluoroscopic or echocardiographic guidance, each model is positioned over the aortic valve. The catheter’s pigtail design encourages stability and makes it easier to pass the aortic valve. The inlet is placed in the LV, whereas the outlet is placed in the ascending aorta [20]. Although widespread, femoral access is not always feasible, such as in the case of severe peripheral artery disease (PAD), which might restrict access in the lower extremities. Alternatively, axillary access can be used in these situations. By providing LV to aorta support, ventricular load is reduced, cardiac output is improved, coronary perfusion pressure and distal flow are increased, and myocardial recovery is enhanced in CS [21,22].

#### 2.1.4. TAH

The TAH is a mechanical support system that replaces the function of the entire heart. Unlike VADs, which assist the ventricles in their action, the TAH completely replaces the valves and ventricles. It instead has two chambers, each with two valves, that are connected to both atria and the great arteries. The first successful human TAH implantation was performed in 1967 by Christiaan Barnard, and it has been used for decades since [23]. Today, TAH is used significantly less often than LVADs, and, unlike LVADs, TAH is not used as destination therapy (DT) in the United States. However, it is still a viable BTT for patients requiring biventricular support [11]. The 1-year mortality of TAH is similar to that of BiVAD, as is the 1- and 5-year post-transplant survival rate. The device is associated with significant complications, with one study showing that 70% of patients experienced major infections, 23% experienced stroke, and 23% experienced major gastrointestinal hemorrhage while on the device [11]. Significantly, some studies have shown that patients with TAH are more likely to need post-implant hemodialysis than patients with BiVAD or LVAD, though analysis of LDH levels between patients who required no dialysis (pre- and post-implant) were not significantly different than LDH levels of those who required only post-implant dialysis [11].

Only one TAH is currently approved for use by the FDA—the Syncardia Total Artificial Heart (Syncardia Systems, Tucson, AZ, USA). A newer TAH, Aeson (Carmat, Vélizy-Villacoublay, France), is currently being studied for FDA approval.

#### 2.1.5. IABP

The IABP has been one of the most frequently used MCS devices since its invention in the 1960s [24]. It has been used to quickly improve hemodynamic stability in patients with CS by means of counterpulsation. When used, a balloon is inserted through the femoral artery and advanced until it is just distal to the left subclavian artery. The IABP is then rapidly inflated and deflated in time with the cardiac cycle. Its inflation during diastole increases diastolic pressure and coronary artery perfusion. Its deflation during the isovolumetric contraction phase of systole decreases afterload, thereby decreasing myocardial oxygen demand and increasing cardiac output [25].

The device is used as a temporary supportive therapy for several clinical problems, including CS (CS), refractory HF, and supportive therapy after coronary artery bypass grafting (CABG) [26]. IABPs are relatively inexpensive, and in theory, they should offer great support to struggling or recently repaired hearts. Some studies have suggested as much; for one of IABPs’ most common uses—hemodynamic support to a patient in CS secondary to MI—one meta-analysis showed that the use of an IABP was associated with lower hospital mortality and lower 30-day mortality compared to no preoperative IABP [26]. Such findings are in the minority, however.

The IABP-Shock II trial, for instance, was a randomized, prospective, multicenter trial that assessed 600 patients for the effects of IABP counterpulsation in the setting of CS with early revascularization. The study showed that IABP use provided no reduction in 30-day, 12-month, or 6-year mortality [27,28]. Another meta-analysis from 2021 analyzed 9 RCTs (1996 patients) comparing the efficacy of IABPs, Impella, and no MCS in the setting of CS or high-risk PCI. They found that there was no difference in 30-day mortality, but that IABP caused increased vascular complications compared to no MCS [29].

Notably, when treating patients with CS using both extracorporeal membrane oxygenation (ECMO) with IABPs, one study showed higher in-hospital survival rates than patients who were treated with ECMO alone (OR = 1.58) [30].

#### 2.1.6. ECMO

ECMO is an MCS device that can be used in different configurations to temporarily support either pulmonary or both pulmonary and circulatory function. ECMO can be performed centrally or peripherally. In peripheral ECMO, both the right femoral artery and the right common femoral vein are cannulated. In central ECMO, the venous cannula is placed in the right atrium, and the arterial cannula is placed in the ascending aorta. ECMO directs blood out of the body and through a membrane oxygenator. The ECMO adds oxygen, removes carbon dioxide, and warms the blood as needed. An extracorporeal pump is also used for circulatory support. In veno-venous ECMO (VV-ECMO), large-bore catheters remove blood from the vena cava and return oxygenated blood to the right heart. Since this method relies on a functioning heart for circulation, it is most often used for cases of reversible respiratory failure. In veno-arterial ECMO (VA-ECMO), the afferent cannula withdraws blood from the vena cava and the right heart, and the efferent cannula returns blood to the aorta, thus bypassing the cardiopulmonary circuit entirely [31].

Despite its use since the 1970s, ECMO has a relative paucity of supportive evidence as to its overall survival benefit. Two of the biggest studies examining ECMO’s use in ARDS—CESAR (2009) and EOLIA (2018)—show mixed reviews. A meta-analysis of their combined RCTs showed a reduction in mortality at 90 days from 48% on standard therapy to 36% with early ECMO therapy [32]. The analysis also concluded that patients with early ECMO were alive and out of the ICU on an average of 8 days earlier than patients on standard therapy. It is worth noting, though, that the treatments for control patients in the CESAR study were not standardized, and that the EOLIA study concluded that there was no difference in 60-day mortality between patients who received early ECMO compared to those who received a standard therapy of mechanical ventilation with ECMO as rescue therapy [33,34]. Additionally, neither study used VV-ECMO [32].

Granted, ECMO has most often been used as an adjunct rescue therapy, and the most common cause of death while on ECMO is multiorgan failure [32]. By the time a patient needs this therapy, they may already have several other comorbidities aside from cardiopulmonary issues that could result in an adverse event. It may be for this reason that ECMO seems to have greater success in severe trauma situations than it does in others. For instance, one study found that patients being treated with ECMO for traumatic injury had a survival rate of 64.7% compared to a rate of 23.5% on conventional mechanical ventilation [35].

### 2.2. Technological Advancements

#### 2.2.1. Enhanced Durability and Reliability

The advancement from pulsatile to continuous flow in LVAD pumps resulted in devices with much greater longevity. Unfortunately, human bodies are designed for pulsatile blood flow, and the use of CF-LVADs resulted in several vascular complications (most commonly, gastrointestinal bleeding) [36]. Some studies show that continuous flow creates a mechanical shear stress that results in acquired von Willebrand Syndrome [37]. The HeartMate 3 overcame this issue by creating pulsatile flow in its CF-LVADs by quickly alternating the speed of the rotor. Combined with HeartMate 3’s magnetically levitated centrifugal motor, this has resulted in a significant reduction in bleeding, stroke, and pump thrombosis [38].

#### 2.2.2. Improved Biocompatibility

An important MCS advancement currently being studied is the Aeson Total Artificial Heart (A-TAH) by Carmat. This device, like Syncardia TAH, is only approved for BTT (in Europe, not the United States). Their ultimate goals for their respective TAHs are approval as DT. The A-TAH, however, has several important features that improve its biocompatibility over older TAHs. Firstly, the A-TAH uses an inner electrohydraulic system to preserve the pulsatility of the native heart, and it uses sensors to adjust heart rate to respond to physiologic demand [39,40]. The pulsatility also helps in the prevention of AVWS [41].

Secondly, the surfaces that contact a patient’s blood are made of a hybrid bovine pericardial tissue processed with glutaraldehyde, which allows for fibrin deposition and endothelialization. This matters because elevation of inflammatory markers after LVAD implantation has been associated with increased mortality, but after 12 months of A-TAH implantation, a study found no difference in pre- and post-implantation inflammatory markers [41,42].

The full impact of this device is yet to be determined, and many studies have yet to be performed, but the A-TAH has the potential to greatly improve the lives of patients requiring TAH or BiVAD. Another TAH currently being studied in vitro is SoftHeart (ETH Zurich, Zurich, Switzerland), which is a pneumatic TAH made almost entirely of silicone rubber. This is the only completely soft TAH developed thus far, and it currently has very limited longevity [43,44,45].

#### 2.2.3. Electrophysiological Impact of MCS

MCS allows for hemodynamic stabilization, particularly in patients in CS due to refractory ventricular arrhythmias and electrical storm. The implantation of MCS devices to ensure end-organ perfusion can partially compensate for the decrease in native cardiac output during arrhythmias. Sometimes, the use of MCS to achieve hemodynamic stabilization may be followed by the spontaneous restoration of sinus rhythm, and other times, electrophysiologic intervention such as catheter ablation is required. Patients with ventricular tachycardia or fibrillation (VT or VF) typically already have reduced left ventricular ejection fraction (LVEF) and lower basal cardiac output, which predispose them to further hemodynamic compromise during catheter ablation due to the anesthesia induction and possible need for recurrent shocks. Through MCS devices, particularly the Impella, essential hemodynamic stabilization can be achieved by maintaining end-organ perfusion, reducing LV wall stress and oxygen consumption, and enabling prolonged mapping during VT episodes [46].

Contrastingly, VT and VF can be seen in the early postoperative period, particularly in the first 30 days after MCS device implantation, and decreasing episodes over the longer term [47]. A retrospective study of 91 patients who underwent LVAD implantation was conducted. Moreover, 35% of patients were noted to have ventricular arrhythmias with de novo monomorphic VT and de novo polymorphic VT or VF after implantation [48]. Interestingly, the risk of ventricular arrhythmias has also been linked to the type of MCS device being used. Although multiple variables are at play, retrospective studies have shown elevated risk of VT and VF after LVAD compared to bi-VAD recipients [47].

## 3. Challenges and Limitations of Current MCS Technologies

### 3.1. Complications

#### 3.1.1. LVADs

Per ESC guidelines, the major nonsurgical complications of LVADs include bleeding, device thrombosis, ischemic and hemorrhagic strokes, renal impairment, multiorgan failure, and infections [49]. Post-LVAD complications can either be early-occurring (<30 days after placement) or late-occurring (>30 days after placement) [49]. The use of antiplatelets and anticoagulants in those with LVAD placement predisposes them to bleeding complications. Early bleeding events within the first 14 days after LVAD placement are typically related to surgery, whereas later bleeding can be caused by the formation of arteriovenous malformations, hepatic dysfunction secondary to right ventricular failure post-LVAD implantation, or even acquired von Willebrand syndrome (AvWS) [50]. Typically, gastrointestinal (GI) bleeding occurs at a median of 33 days from surgery, with the greatest risk being within the first postoperative month [50]. Possible explanations for GI bleeding post-LVAD implantation include the development of angiodysplasia due to increased shear stress and intraluminal pressure or the development of AvWS from red blood cell mechanical damage, causing von Willebrand factor (vWF) deformity [50].

Despite antiplatelet and anticoagulation therapy, thromboembolic events are a common complication of LVAD therapy, with neurologic events being one of the primary causes of death [51]. Pump thrombosis remains the leading cause of device malfunction and embolic strokes. Studies have shown that the target INR of 2.6 is optimal to avoid thrombotic and bleeding complications, supporting the current practice of a target INR range being 2.0 to 3.0 [52].

Infections, another common complication of LVAD implantation, remain the second leading cause of death in patients who survive the first six months of LVAD support. Rates of infections related to LVADs range from 30% to 50%. These infections include pneumonias and sepsis, followed by driveline site infections (DLIs), and pump interior and pocket infections. When comparing HeartWare and HeartMate II, infections are lower in HeartWare patients, likely because this device is implanted within the pericardial space without requiring a pocket pump [53]. Unfortunately, for DLIs, no formal guidelines exist regarding the initial antibiotics to use or the length of antibiotic therapy required for treatment. If initial antibiotic therapy for at least two weeks is not sufficient, then surgical debridement or prolonged intravenous antibiotic therapy may be required. Worsening clinical status despite these therapies may indicate a possible fungal infection for which antifungal therapy should be considered, given the high mortality risk [54].

Aortic insufficiency (AI) is frequently seen in LVAD-supported patients, ranging from 11% to as high as 42%. The pathophysiologic mechanism of LVAD-induced AI occurs from the incorrect angle of blood outflow from the LVAD into the ascending aorta, increasing the risk of aortic root dilation, aortic wall changes due to shear stress, aortic valve injury and degeneration, and overall aortic root weakening. Once AI develops in an LVAD-supported patient, the valvular incompetence lowers the LVAD’s pump efficiency, leading to worsening HF [55]. Of note, the development of AI in patients with the HeartMate II LVAD is heavily covered in published studies, which contrasts with the rarity of AI development in patients with HeartWare LVAD [56].

Lastly, right ventricular failure (RVF) can occur after LVAD implantation due to increased cardiac output, which may overwhelm the load-sensitive right ventricle (RV). The RV body also becomes altered due to the shifting of the interventricular septum once the left intraventricular pressure falls due to the LVAD [57].

#### 3.1.2. BiVADs

Patients requiring BIVAD support are typically more critically ill. Per the INTERMACS database, where adverse events and outcomes were recorded for primary implants of LVADs or BiVADs, patients who required BiVAD therapy experienced a lower survival rate and higher adverse event rate compared to patients supported with LVAD alone. Adverse events were significantly higher in terms of infection, bleeding, neurologic events, and device failure in the BiVAD group compared to the LVAD group [13]. Other adverse event profiles include renal dysfunction and device malfunction, regardless of whether the RVAD was temporary or durable [58].

#### 3.1.3. Impella

Adverse hemorrhagic and ischemic events contribute to the bleeding and vascular complications associated with the Impella. The need for large-bore access sites, anticoagulation, ongoing shock-induced coagulation dysfunction, and device-related hemolysis from mechanical shear forces largely contributes to these complications. The Italian IMP-IT registry found that Impella-related complications were higher in the CS patients receiving Impella therapy in comparison to those with high-risk percutaneous coronary intervention (HR-PCI) [59]. Common complications of Impella use include major bleeding at access sites, limb ischemia, and hemolysis [19].

#### 3.1.4. TAH

**Figure 1:**
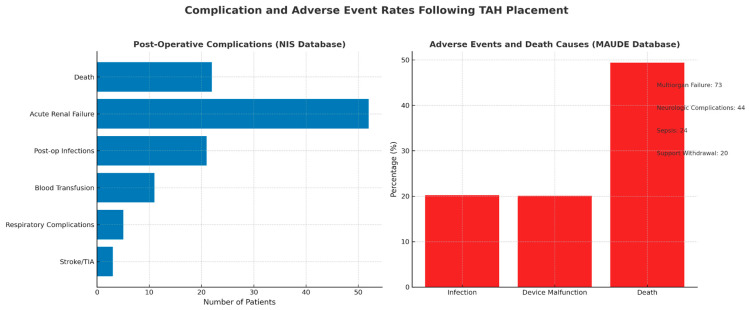
Postoperative and adverse event rates after TAH placement based on NIS and MAUDE database reports.

A meta-analysis of TAH utilization studying 75 patients from the NIS administration database who underwent TAH (Figure 1) found that the postoperative complication rate was *n* = 22 for death, *n* = 52 for acute renal failure, *n* = 21 for postoperative infections, *n* = 11 for required blood transfusions, *n* = 5 for respiratory complications, and *n* = 3 for stroke/transient ischemic attack (TIA) [60]. Another study identified 453 patients who received TAH in the MAUDE database (Figure 1) and noted that the most common adverse event reported was infection at 20.2% followed by device malfunction at 20.1% [61]. Death was reported in 49.4% of all reported cases (*n* = 224/453), and the most common cause was multiorgan failure *n* = 73, followed by neurologic complications like cerebrovascular accident (CVA) *n* = 44, sepsis *n* = 24, and support withdrawal *n* = 20 [61].

#### 3.1.5. IABP

**Figure 2:**
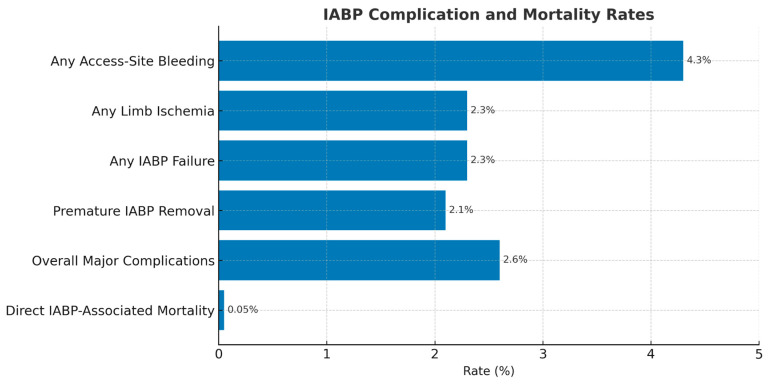
IABP-associated complication and mortality rates. Shown are events with an incidence greater than 2%, the overall major complication rate (2.6%), and direct IABP-related mortality (0.05%). The IABP-SHOCK II trial reported no significant difference in complication rates between IABP and control groups (all *p* > 0.05) [63].

The most common complications associated with IABP include vascular complications such as stroke and limb ischemia. An incidence of 2.6% for major complications is noted in the benchmark registry [62]. These significant complications include severe limb ischemia, severe bleeding, balloon leaks, or death [62]. However, the overall IABP-associated mortality was about 0.05% [62]. The benchmark registry found that independent risk factors for major complications were female gender, peripheral vascular disease, body surface area less than 1.65 m^2^, and age ≥ 75 years [63]. Additional risk factors to compound the ones listed include duration of required IABP support, size of catheter used, previous history of diabetes mellitus, and a low cardiac index ≤ 2.2 L/min [63]. The IABP-SHOCK II trial found no significantly higher risk of complications in those receiving IABP compared to the control group [63]. These results are depicted in Figure 2 below.

#### 3.1.6. ECMO

Morbidity associated with ECMO use is significantly high, with the most common complications being bleeding, thrombosis, hemolysis, CVA, and limb ischemia. Given the mechanism by which cannulation and oxygenation are performed, there are instances of significant blood contact against foreign objects and surfaces, stasis of blood in the heart, and increased risk of disseminated intravascular coagulation (DIC); the risk and incidence of thrombosis while on VA-ECMO is substantial. These risks warrant therapeutic anticoagulation therapy during ECMO [64]. However, due to consumption of clotting factors and thrombocytopenia, the incidence of bleeding with therapeutic anticoagulation remains high, with about 42% of patients requiring re-exploration surgery [64]. Heparin-induced thrombocytopenia, in addition to platelet injury throughout the circuit, contributes to the high incidence of bleeding. High-risk bleeding tends to occur in multiple organs, but most commonly at the cannulation sites, the brain, and the gastrointestinal tract. CVA. Similarly, the incidence of CVA is reported to be up to 3.3–17.6% for ischemic stroke and 1.6–5% for hemorrhagic stroke [65]. Limb ischemia can be a major complication of peripheral VA-ECMO, where femoral artery cannulation is used, causing upwards of 10% of cases requiring treatment with fasciotomy and about 5% requiring treatment with amputation [66].

Some complications are specific to VA-ECMO, such as LV distension and Harlequin syndrome. The LV distension develops secondary to the retrograde flow of oxygenated blood in the ascending aorta, which increases the load against which the failing LV has to eject. The strong current of retrograde flow of blood can lead to the development of pulmonary edema and, in some cases, cardiac thrombi [67]. Sometimes, if LV distension and concern for intracardiac stasis are present, an LV vent can be inserted.

**Figure 3:**
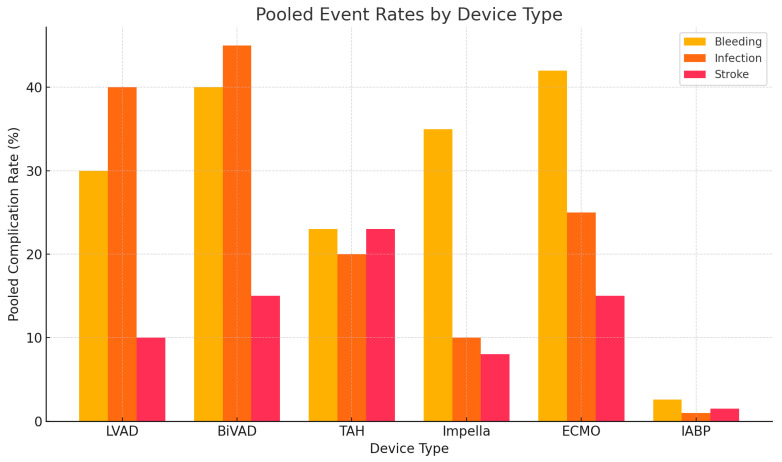
Pooled complication rates for major adverse events associated with mechanical circulatory support (MCS) devices. This figure summarizes pooled rates of bleeding, infection, and stroke for major MCS devices based on data from the review.

Due to the mixing of anterograde ejected deoxygenated blood, such as in the case of respiratory failure, and retrograde flow of oxygenated blood, a mixing cloud or zone forms. This mixing cloud is exposed to the coronary and cerebral circulations. This issue typically arises as a result of LV function recovery, where the mixing cloud is found in the descending aorta, past the cerebral and coronary arterial outflow. Over time, exposure to deoxygenated blood in the vital organs poses increased harm and risk of mortality [68]. Figure 3 below illustrates pooled event rates by different device types.

### 3.2. Limited Long-Term Data

#### 3.2.1. LVAD Long-Term Survival Outcomes and Device Performance

**Figure 4:**
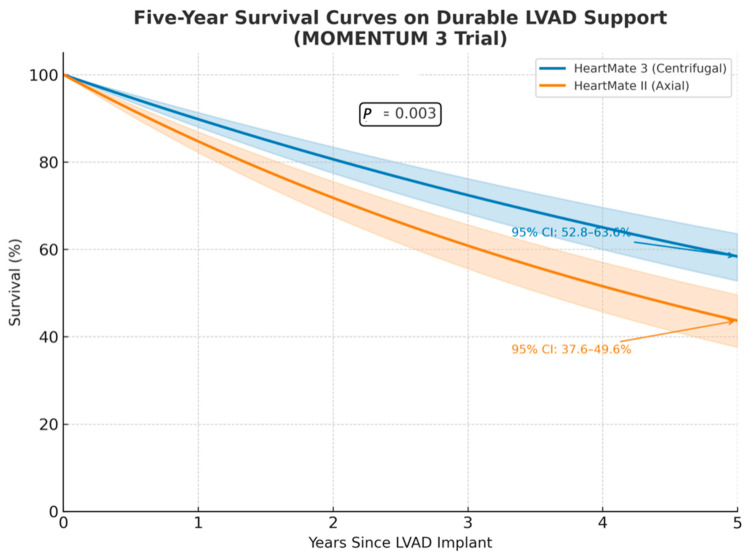
The five-year survival on continuous-flow durable left ventricular assist device (LVAD) support from the MOMENTUM 3 trial. Five-year survival was 58.4% (95% CI: 52.8–63.6) in the centrifugal flow pump group (HeartMate 3) and 43.7% (95% CI: 37.6–49.6) in the axial flow pump group (HeartMate II) [71].

Survival with durable MCS has significantly improved over the last two decades. The current 1-year survival rate is about 87%, with an absolute improvement in this rate of about 35% over 18 years. A 2001 study, Randomized Evaluation of Mechanical Assistance for Treatment of Congestive Heart Failure (REMATCH), found a 1-year survival rate of 52% when compared against medical management alone [69]. A newer study called Multicenter, Study of MagLev Technology in Patients Undergoing MCS Therapy with HeartMate 3 (MOMENTUM 3) found a 1-year and five-year survival rate of 84% and 52% with use of the HeartMate 3 LVAD, respectively, improved from the 1-year and five-year survival rate of 82% and 44% with use of the HeartMate II LVAD, respectively [69]. This can also be seen in Figure 4 below. This study, along with data from the Society of Thoracic Surgeons Intermacs National Database (STS Intermacs) validated the survival benefits of the HeartMate 3 device [70].

In addition to survivability, improvement in patient-reported outcomes such as quality of life and functional capacity per New York Heart Association (NYHA) functional class was noted with a reduction in anxiety/depression and hospital admissions, proving the impact of durable MCS [69]. MOMENTUM 3 found that 95% of the selected patients for HeartMate 3 were classified as NYHA Class IV before device implantation, and 77% experienced improvement to NYHA Class I or II within six months of MCS therapy. Improvement in the six-minute walk test was evidenced by an overall increase in distance walked from 140 m to 230 m post-device implantation [69].

#### 3.2.2. BIVAD Long-Term Survival Outcomes and Device Performance

Literature demonstrates that the overall 1-year survival in patients supported with BiVAD therapy is significantly lower compared to LVAD-only cohorts at about 50% vs. 83% [58]. The survival of concurrent BiVAD-supported patients was higher than those who received sequential VAD therapy, LVAD placement followed by a separate surgical encounter for RVAD placement, at about 56% compared to 42%, respectively [58]. Pre-implant patient characteristics were controlled through a propensity-score matching analysis study and factors studied included age, sex, race, BMI, implant brand, implant year, diagnoses, inotrope status, valvular abnormalities of mitral and tricuspid regurgitation, ECMO usage within 48 h of implant, and various other laboratory values like bilirubin and albumin. This study supports the avoidance of a sequential RVAD placement unless it is necessary [58].

#### 3.2.3. Impella Long-Term Survival Outcomes and Device Performance

A prospective, multicenter observational study evaluating 804 patients receiving Impella for CS found that patients who received Impella 5.5 alone, compared to multiple types of MCS, had an overall favorable in-hospital, six-month, and 12-month survival rate, whether they were bridged to native heart survival or became candidates for Htx [72]. Many small retrospective studies have been performed comparing the Impella to IABP. These include the ISAR-SHOCK trial, which found that Impella improved hemodynamics in patients but not mortality, and the IMPRESS trial, which found that there was no difference in 30-day (48.5% for Impella vs. 46.4% for IABP, *p* = 0.64) or 6-month mortality between CS patients receiving IABP versus Impella [73,74]. Notably, the Impella group experienced higher rates of severe bleeding and peripheral vascular complications [75].

#### 3.2.4. TAH Long-Term Survival Outcomes and Device Performance

A multicenter, retrospective analysis of about 217 patients undergoing TAH implantation between 2014 and 2019 in six different American medical centers studied the survival and adverse event profile. This study found that 63.5% of patients with TAH successfully transitioned to heart transplant, while 34.5% of patients died before heart transplant [76]. Overall patient survival in the TAH-receiving cohort at the 1-year, 2-year, and 5-year mark was 75%, 64%, and 58%, respectively. Of the 63.5% of patients transplanted, the overall survival at the 6-month, one-year, two-year, and five-year mark was 88%, 84%, 79%, and 74%, respectively [76]. This study concluded that nearly two-thirds of the patients receiving TAH could successfully undergo heart transplantation with an acceptable range of overall survival and survival post-transplant among these critically ill individuals [76].

#### 3.2.5. IABP Survival Outcomes and Device Performance

The IABP-SHOCK II multicenter, randomized, open-label trial studying 600 patients with acute STEMI complicated by CS confirmed the lack of benefit of IABP on overall clinical outcomes when comparing mortality at 30 days in the control group. A follow-up retrospective study was performed on data collected from the European Registry on Patients with ST-Elevation MI Transferred for Mechanical Reperfusion, from which 1650 consecutive patients were located in an attempt to reassess IABP-use outcomes in “unselected” patients from the IABP-SHOCK II trial [77]. Notably, 51 patients with CS on initial admission were identified and stratified by IABP use or not during hospital stay. Notably, 30 of the 51 patients elected underwent IABP placement, while the remaining 21 did not receive IABP placement [77]. This study found that even after adjustment for age and sex, there was no difference in short- and long-term mortality between patients treated with and without IABP [77].

RHF significantly worsens the prognosis in patients who do not benefit from IABP therapy. The presence of RVD is associated with higher mortality and highlights the limitations of IABP in managing biventricular failure. Early recognition of RHF and consideration of alternative mechanical support strategies are crucial for improving outcomes in this patient population [78].

#### 3.2.6. ECMO Survival Outcomes and Device Performance

**Figure 5:**
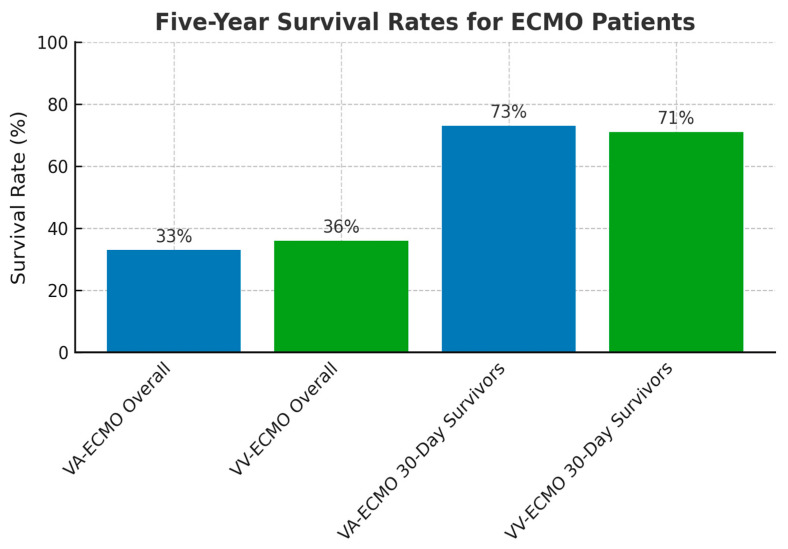
Overall survival beyond five years was slightly higher in the VV-ECMO group (36%) compared to the VA-ECMO group (33%). Patients who survived the first 30 days after ECMO initiation demonstrated markedly improved long-term outcomes in both groups.

Long-term survival and outcomes after ECMO remain limited in the literature. Per the Extracorporeal Life Support Organization (ELSO) Live Registry Dashboard, in the last five years, of all 111,129 patients undergoing VA or VV-ECMO in the world, 53% survived to transfer or discharge [79]. A single-center, retrospective chart review-based survival analysis over a period of five years was performed in 2023 to evaluate the five-year survival and overall quality of life in patients treated with ECMO, both VV and VA. Notably, 370 patients, 288 on VA-ECMO and 82 on VV-ECMO, found that the over five-year survival was 33% for VA-ECMO patients and 36% for those on VV-ECMO [80]. It was noted that in patients who survived the first 30 days after ECMO initiation, the five-year survival rate significantly improved to 73% on VA-ECMO and 71% on VV-ECMO (Figure 5) [80].

About 56% of the patients studied participated in a median follow-up of 4.2 years and 5.7 years after VA-ECMO and VV-ECMO, respectively. Notably, 29% of VA-ECMO survivors and 75% of VV-ECMO survivors reported difficulties with activities of daily living (ADLs). Overall, these findings suggest that among patients who survive the critical early post-ECMO period, long-term survival and functional outcomes are generally acceptable [80]. Comparative data for all the MCS devices can be found below in Table 1.

### 3.3. Cost and Accessibility

Mechanical circulatory support device use comes with a high-cost burden. Significant differences in cost were noted between devices like ECMO and Impella in comparison to IABP. The adjusted median standardized cost of hospitalization was highest with ECMO $210383.0 (IQR: $165,760–$239,373), followed by Impella $142,518 (IQR: $126,845–$179938) and IABP $132,059.7 (IQR: $113,794–$160,243.7) [81]. The adjusted cost of hospitalization was also highest amongst patients receiving ECMO support, with a mean of $210,383 ± $66,028) [81]. During the research period, it was noted that the cost of IABP and ECMO reduced with time, but the cost associated with Impella remained the same. Unfortunately, a direct relationship was found between cost-adjusted hospitalization amount and device-associated stroke and bleeding. However, a direct relationship between 30-day mortality and overall price-adjusted hospitalization cost was not observed. After adjusting for patient factors, only patients treated at the highest-cost-adjusted hospitals showed a lower 30-day mortality rate [81].

## 4. Future Directions in Mechanical Circulatory Support

### 4.1. Next-Generation Devices

#### 4.1.1. Fully Implantable and Wearable Devices

Recent advancements in fully implantable, wearable LVADs have made significant strides in addressing the challenges faced by HF patients requiring long-term mechanical support. These devices offer a substantial benefit by eliminating the need for external drivelines, which are a major source of infection and complications in conventional models. In fact, driveline infections affect up to 40% of patients with traditional models, presenting a significant obstacle to their long-term success [10]. By fully implanting the device, this risk is minimized, leading to improved patient outcomes. Advances such as the HeartMate 3 have demonstrated improved survival and lower thrombotic complication rates compared to earlier devices, further validating the move toward next-generation designs [10].

However, creating a fully implantable model introduces its challenges. One of the biggest obstacles is ensuring a reliable power supply without external wires. Recent progress in battery technology and wireless power transmission systems has enabled reductions in the size and weight of these devices, though long-term battery durability remains a concern [82]. Furthermore, although these devices aim to replicate natural heart function, they must withstand mechanical stress over prolonged periods. The occurrence of pump thrombosis, in which blood clots form within the pump, remains a significant issue, with an annual incidence of 5 to 10 percent [10]. Long-term durability and the minimization of complications such as device failure and thromboembolic events are vital considerations in the further development of fully implantable circulatory support devices.

#### 4.1.2. Bioengineered and Biological Solutions

One promising development is the integration of bioengineered materials aimed at enhancing biocompatibility, reducing thrombosis, and promoting tissue integration. Research into bioengineered tissues and scaffolds made from natural polymers has shown promise in reducing thromboembolic events and infection rates. These approaches aim to improve endothelialization and device integration with natural tissues, ultimately reducing complications like pump thrombosis. Although pump positioning has been identified as a significant factor influencing thrombosis risk in devices such as the HVAD, the development of bioengineered surfaces offers a complementary strategy to further mitigate thrombosis [83]. Improper positioning of the pump, particularly in HVAD devices, has been associated with a higher incidence of thrombosis, highlighting the role of imaging in long-term management [83].

Advances in tissue engineering have also led to the conceptualization of “living devices,” which combine mechanical support with regenerative capabilities. By using stem cells, scaffolds, and bioactive materials, researchers have developed systems that could stimulate myocardial repair and promote tissue healing. In animal models, stem cell-based devices have been shown to improve myocardial function by reducing fibrosis and promoting regeneration [10]. These bioengineered devices hold promise for providing both mechanical support and biological restoration, potentially improving long-term outcomes.

#### 4.1.3. AI and Machine Learning Integration

Artificial intelligence and machine learning are expected to revolutionize patient care by enabling continuous monitoring and predictive analytics for device performance. Machine learning algorithms can analyze vast amounts of data from device sensors, detecting subtle changes in patient status that could indicate impending complications. A study by Malone et al. found that AI-assisted monitoring can predict the onset of pump thrombosis with a sensitivity of 85% and a specificity of 90% [82]. Early detection capabilities such as these allow for timely interventions, reducing the risk of life-threatening events like stroke or pump failure.

Additionally, AI systems are increasingly used to personalize patient care. Machine learning algorithms can help tailor anticoagulation therapy by predicting optimal anticoagulant dosages based on individual patient profiles [82]. This personalized approach could reduce the incidence of bleeding and thrombotic complications, which remain significant challenges in LVAD management. By combining continuous monitoring with predictive analytics, AI-driven management strategies may significantly improve survival rates and enhance quality of life for patients [10].

### 4.2. Stem Cell Therapy and Regenerative Approaches

Recent advancements in regenerative medicine have highlighted the potential of stem cell-derived therapies for heart failure. Li et al. developed a multilayered cardiac tissue using human-induced pluripotent stem cell-derived cardiomyocytes (hiPSC-CMs) seeded onto a fibrous scaffold [84]. This engineered tissue achieved a thickness of approximately 1 mm and demonstrated enhanced contractile properties and cytokine secretion compared to traditional single-layer constructs. When applied to a rat model of myocardial infarction, the multilayer group showed improved functional recovery and less fibrosis [84]. These results indicated that the appropriate hiPSC-CM dose requires careful evaluation in developing clinical therapy.

These findings underscore the promise of hiPSC-CM-based therapies in cardiac regeneration. However, further large-scale, randomized controlled trials are necessary to validate the long-term safety and effectiveness of these approaches in human patients.

### 4.3. The Role of Gene Therapy

Gene therapy is emerging as a promising approach for treating heart failure by targeting underlying genetic and molecular mechanisms. Khan et al. investigated the therapeutic potential of adeno-associated virus serotype 9 (AAV9)-mediated delivery of cardiac bridging integrator 1 (cBIN1) in a canine model of chronic heart failure due to ischemic cardiomyopathy [85]. In this study, adult mongrel dogs underwent ligation of the left anterior descending artery to induce heart failure. Once the LVEF dropped below 40%, the animals received a one-time series of endocardial injections of either AAV9-cBIN1 or AAV9-GFP (control). The AAV9-cBIN1-treated group showed significant improvement in LVEF (from 29 ± 3% to 42 ± 2%; *p* = 0.0095) and global longitudinal strain (from −7.1 ± 0.9% to −12.5 ± 1.6%; *p* = 0.0095) compared to the control group [85]. Additionally, treated animals exhibited improved T-tubule morphology, reduced left ventricular chamber size, favorable plasma biomarker profiles, and enhanced endocardial voltage [85]. Importantly, all animals in the AAV9-cBIN1-treated group survived the study period, highlighting the therapy’s potential efficacy and safety [85].

These findings underscore the potential of gene therapy as a transformative treatment for heart failure, offering hope for improved patient outcomes through targeted molecular interventions.

### 4.4. Combining MCS with Other Therapies

The integration of mechanical circulatory support with adjunctive therapies, such as pharmacological agents and gene therapies, offers a multifaceted approach to optimizing patient outcomes. For instance, combining mechanical support with gene therapies promoting angiogenesis has demonstrated improvements in myocardial perfusion and trends toward better ventricular function [86].

Additionally, targeting immune responses through innovative therapies holds promise for reducing infection rates, a leading cause of morbidity and mortality in LVAD recipients. Fu et al. discussed how immune modulation strategies could decrease infection-related complications, although precise figures regarding infection reduction rates are still under investigation [87].

Another promising avenue is combining mechanical support with novel pharmacological agents. Sodium-glucose cotransporter 2 inhibitors have emerged as effective treatments for HF, improving outcomes and potentially reducing the need for advanced mechanical support if initiated early [88]. In addition to improving glycemic control, SGLT2 inhibitors have demonstrated cardiovascular benefits even in patients without diabetes [88]. Their mechanisms include reducing myocardial fibrosis, improving ventricular remodeling, and decreasing HF hospitalizations. Early initiation of SGLT2 inhibitors after the diagnosis of HF has been associated with better preservation of cardiac function, highlighting the importance of integrating pharmacologic strategies alongside mechanical circulatory support [88]. As research evolves, these combined strategies may offer synergistic benefits, improving both device longevity and patient quality of life.

### 4.5. Xenotransplantation

An alternative to cardiac transplantation, xenotransplantation, particularly porcine-to-human heart transplantation, is up and coming. Due to advances in somatic cell nuclear transfer technology (i.e., cloning) and CRISPR/Cas9 gene editing, the removal of immunogenic epitopes and porcine endogenous retroviruses has been enabled, reducing the risk of hyperacute rejection [89]. Specifically, thus far, gene editing has been used to modify 10 genes in a domestic pig, making it a viable alternative to conventional heart transplantation, albeit temporarily. Xenotransplantation may eventually be used as a destination therapy for patients who are not eligible for lasting MCS, as well as a bridge to transplant for patients waiting for human hearts, as bioengineering and immunomodulation progress. Notwithstanding moral and legal challenges, its advancements make it a viable avenue for advanced heart failure treatment in the future.

## 5. Conclusions

Advances in MCS therapies have expanded the available treatment options for patients with severe HF and CS. These devices have unique roles in providing bridging therapy for recovery, heart transplant, or even long-term support. The overall use of MCS devices is on the rise, particularly the Impella device, followed by ECMO, with LVADs notable for their use as BTT and, due to their efficacy, as destination therapy. Despite an increase in MCS device use, there has not been a significant difference noted in 30-day mortality or hospitalization rates for each device type, which suggests that increased use does not necessarily equate to improved patient outcomes. While strong evidence is available to suggest overall improvement in survival outcomes with MCS therapies like LVADs, Impella, and ECMO in critically ill patients who survive the first 30 days post-implantation of MCS devices, the high rates of complications of bleeding and thrombosis secondary to these therapies remain a significant challenge. Cost considerations and improvement in overall quality of life are important considerations for the use of MCS therapies, highlighting the importance of careful patient selection, a multidisciplinary medical team approach, and long-term follow-up.

## Figures and Tables

**Table 1:** Comparative Summary Table of Mechanical Circulatory Support Devices.

Device	Primary Indications	Common Complications	Survival Outcomes	Typical Patient Population
LVAD	- Refractory left-sided HF - Bridge to transplantation - Destination therapy	- Bleeding (GI, perioperative) - Thrombosis - Stroke - Infection (driveline, pump) - RV failure - Aortic insufficiency	- 1 year: ~84–87% - 5 years: ~52% - Improved QoL (NYHA class, walk test)	- Patients with end-stage HF not eligible for transplant or are awaiting transplant
BiVAD	- Biventricular HF - Bridge to transplant	- Higher rate of infection - Bleeding - Neurologic events - Device failure - Renal dysfunction	- 6 months: ~56% - 1 year: ~50% - Concomitant implant is better compared to sequential implant	- Critically ill patients with RHF + LHF, often not candidates for isolated LVAD
TAH	- Replacement for both ventricles - Bridge to transplant (not a destination therapy in U.S.)	- Infection - Device malfunction - Renal failure - Stroke	- 1 year: 75% - 2 years: 64% - 5 years: 58% - 63.5% successfully transitioned to heart transplant	- Patients with end-stage biventricular HF unsuitable for LVAD/BiVAD and awaiting transplant
Impella	- Cardiogenic shock - High-risk PCI	- Bleeding - Hemolysis - Limb ischemia	- 1 month: ~48% - No survival benefit over IABP	- Acute cardiogenic shock patients during/after intervention
IABP	- Cardiogenic shock, especially post-MI - Supportive therapy post-CABG	- Limb ischemia - Stroke - Balloon leak - Bleeding	- Overall, IABP mortality ~0.05%	- Used for transient support in patients with ischemic cardiogenic shock or low output post-surgery
ECMO (VA/VV)	- VA: cardiogenic shock, cardiac arrest - VV: refractory ARDS	- Bleeding - Thrombosis - Stroke - Hemolysis - Limb ischemia - LV distension	- 5 years (overall): ~33% (VA) and ~36% (VV) - 5 years (after first 30-day survival): ~73% (VA) and ~71% (VV)	- Patients in severe respiratory/circulatory failure or multiorgan dysfunction

Summary of all mechanical circulatory support devices along with their indications, complications, survival, and patient population. LVAD: Left ventricular assist device, BiVAD: Biventricular assist device, TAH: Total artificial heart, IABP: Intra-aortic balloon pump, ECMO: Extracorporeal membrane oxygenation, VA: veno-arterial, VV: veno-venous, HF: heart failure, GI: gastrointestinal, RV: right ventricle, QoL: Quality of Life, NYHA: New York Heart Association, RHF: right heart failure, LHF: Left heart failure, PCI: percutaneous coronary intervention, MI: myocardial infarction, CABG: coronary artery bypass graft.
